# Systems Analysis of MVA-C Induced Immune Response Reveals Its Significance as a Vaccine Candidate against HIV/AIDS of Clade C

**DOI:** 10.1371/journal.pone.0035485

**Published:** 2012-04-19

**Authors:** Carmen Elena Gómez, Beatriz Perdiguero, Victoria Jiménez, Abdelali Filali-Mouhim, Khader Ghneim, Elias K. Haddad, Esther D. Quakkerlaar, Julie Delaloye, Alexandre Harari, Thierry Roger, Thomas Dunhen, Rafick P. Sékaly, Cornelis J. M. Melief, Thierry Calandra, Federica Sallusto, Antonio Lanzavecchia, Ralf Wagner, Giuseppe Pantaleo, Mariano Esteban

**Affiliations:** 1 Department of Molecular and Cellular Biology, Centro Nacional de Biotecnologia, CSIC, Madrid, Spain; 2 Vaccine and Gene Therapy Institute of Florida, Port St. Lucie, Florida, United States of America; 3 Department of Immunohematology and Blood Transfusion, Leiden University Medical Center, Leiden, the Netherlands; 4 Infectious Diseases Service, Department of Medicine, Centre Hospitalier Universitaire Vaudois and University of Lausanne, Lausanne, Switzerland; 5 Division of Immunology and Allergy, Centre Hospitalier Universitaire Vaudois, Lausanne, Switzerland; 6 Institute for Research in Biomedicine, Bellinzona, Switzerland; 7 University of Regensburg, Regensburg, Germany; University of Cape Town, South Africa

## Abstract

Based on the partial efficacy of the HIV/AIDS Thai trial (RV144) with a canarypox vector prime and protein boost, attenuated poxvirus recombinants expressing HIV-1 antigens are increasingly sought as vaccine candidates against HIV/AIDS. Here we describe using systems analysis the biological and immunological characteristics of the attenuated vaccinia virus Ankara strain expressing the HIV-1 antigens Env/Gag-Pol-Nef of HIV-1 of clade C (referred as MVA-C). MVA-C infection of human monocyte derived dendritic cells (moDCs) induced the expression of HIV-1 antigens at high levels from 2 to 8 hpi and triggered moDCs maturation as revealed by enhanced expression of HLA-DR, CD86, CD40, HLA-A2, and CD80 molecules. Infection *ex vivo* of purified mDC and pDC with MVA-C induced the expression of immunoregulatory pathways associated with antiviral responses, antigen presentation, T cell and B cell responses. Similarly, human whole blood or primary macrophages infected with MVA-C express high levels of proinflammatory cytokines and chemokines involved with T cell activation. The vector MVA-C has the ability to cross-present antigens to HIV-specific CD8 T cells *in vitro* and to increase CD8 T cell proliferation in a dose-dependent manner. The immunogenic profiling in mice after DNA-C prime/MVA-C boost combination revealed activation of HIV-1-specific CD4 and CD8 T cell memory responses that are polyfunctional and with effector memory phenotype. Env-specific IgG binding antibodies were also produced in animals receiving DNA-C prime/MVA-C boost. Our systems analysis of profiling immune response to MVA-C infection highlights the potential benefit of MVA-C as vaccine candidate against HIV/AIDS for clade C, the prevalent subtype virus in the most affected areas of the world.

## Introduction

While a vaccine against HIV/AIDS has remained elusive since the pandemic first appeared, the recent findings from the phase III Thailand clinical trial with the combination of a canary poxvirus vector (ALVAC) and purified protein gp120 (RV144) giving 31.2% protection against HIV infection [1], while it was not sustain, it open the possibility of improving HIV/AIDS efficacy through modification of some of the vaccine components similar as those used in the Thai trial. Potential vaccine improvements include the use of other attenuated poxvirus vectors, like MVA and NYVAC [2], [3], genetic modification of the poxvirus vectors through deletion of viral immunomodulatory genes [4], [5], [6], [7], [8] and prime/boost combination with heterologous vectors with or without adjuvants and co-stimulatory molecules [9], [10], [11]. A number of phase I/II clinical trials have been performed or are on-going with poxvirus vectors administered alone or in prime/boost combination (http://www.iavi.org). The clinical findings obtained thus far revealed that attenuated poxvirus vectors when used alone induced lower immune responses that when combined in prime/boost protocols with heterologous vectors [9], [12]. As yet it remains unclear which of the poxvirus vectors is optimal in triggering immune responses. A deep understanding on the immune characteristics of these vectors is needed, as each vector might impact the immune system differently. This has been highlighted for NYVAC and MVA vectors [13], [14], as these vectors have differential deletions of viral immunomodulatory genes. One of the attenuated poxvirus vectors that is going to enter phase I clinical trials is based on MVA expressing HIV antigens for subtype C, i.e, Env gp120 as a cell released product and the fusion polyprotein Gag-Pol-Nef as intracellular component (referred as MVA-C). As yet, there is limited information on the characteristics of this vector. Herein, we use systems biology to characterize the global immune response and provide in-depth characterization of the biological and immune features of MVA-C in human cells and in animals. Our results showed that MVA-C is an excellent immunogen, as it expresses at high levels the HIV-1 antigens in human moDCs, triggers DC maturation, activates wide expression of immunostimulatory molecules, induces cross-presentation and CD8^+^ T cell proliferation and, in vaccinated mice by prime/boost protocols, activates HIV-1-specific CD4 and CD8 memory responses that are polyfunctional and with effector memory phenotype (TEM). These observations point MVA-C vector as promising HIV/AIDS vaccine candidate.

## Results

### Expression of HIV antigens in DCs infected with MVA-C

**Figure 1:**
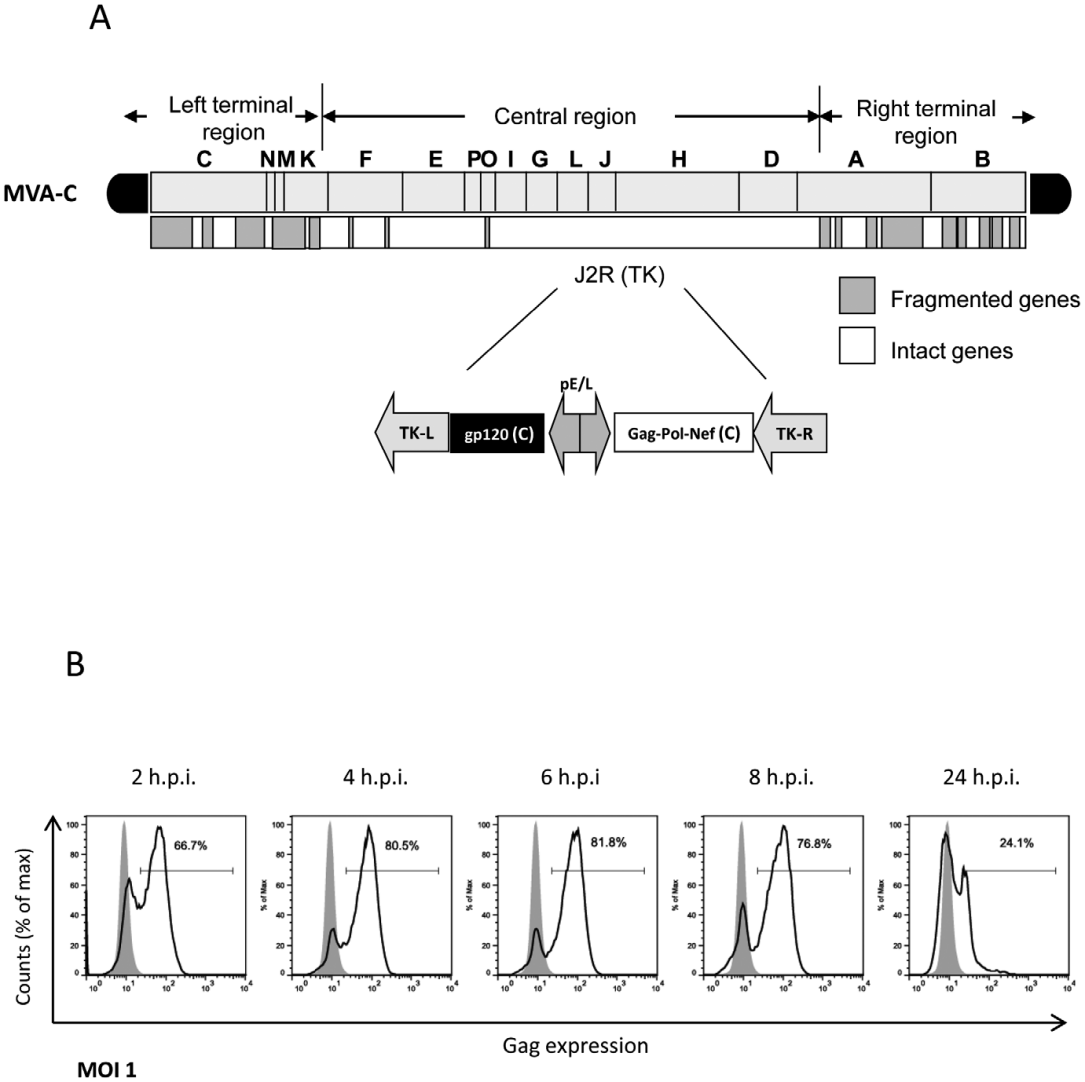
MVA-C induces the expression of HIV gag antigen early after infection of human DCs. A) Schematic diagram of MVA-C genome. B) Histograms show anti-Gag KC57 staining of infected moDCs. Cells were infected at 1 pfu/cell and at different times after infection cells were harvested and stained for Gag expression by ICS as described under Materials and Methods. Percentage of Gag-expressing cells is indicated.

We have previously described that MVA-C infection of human HeLa cells in culture induces the expression of gp120 and of the polyprotein Gag-Pol-Nef as determined by Western blot [15]. The organization of the HIV genes in the TK locus of the MVA genome is shown as scheme in Fig. 1A. To define the gene expression capacity of MVA-C in human monocyte-derived dendritic cells (moDCs), we evaluated the expression levels of HIV Gag by flow cytometry. As shown in Fig. 1B, Gag expression increases with time of infection. Of relevance, 60% of the cells expressed Gag by 2 h post infection and the levels were reduced by 24 h, possibly due to apoptosis induction at late times post infection [16]. The high levels of HIV antigen expression in moDCs early in infection is due to the nature of the promoter, as both gp120 and Gag-Pol-Nef genes expression is driven by a synthetic early/late virus promoter. These findings showed the high capacity of MVA-C to express HIV antigens in human DCs, which are critical cells in the activation of innate immune responses.

### Transcriptional analyses of mDC and pDC infected with MVA-C revealed the global induction of innate and adaptive immunoregulatory pathways

**Figure 2:**
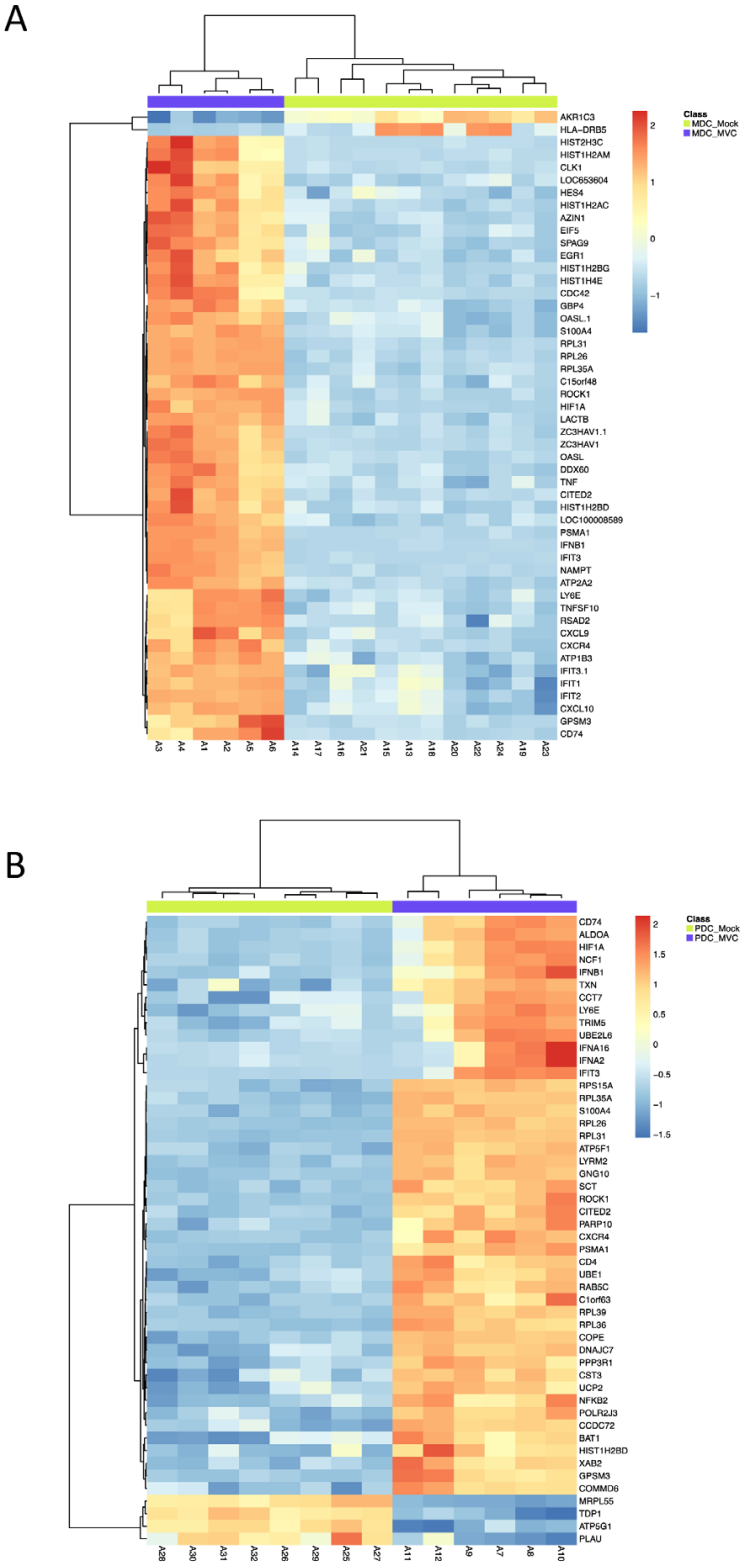
Genes induced by MVA-C in human mDCs and pDCs. A) Gene expression heatmap using the top 50 differentially expressed genes resulting from comparing myeloid DCs infected with MVA-C (MDC_MVC) and mock-infected (MDC_Mock) groups. B) Gene expression heatmap using the top 50 differentially expressed genes resulting from comparing plasmacytoid DCs infected with MVA-C (PDC_MVC) and mock-infected (PDC_Mock) groups. Genes selected as differentially expressed based on the following criteria: adjusted p-value<0.05 and |FC|>1.3. The scale shows the level of gene expression where Red and Blue correspond to up- and down-regulation respectively.

**Figure 3:**
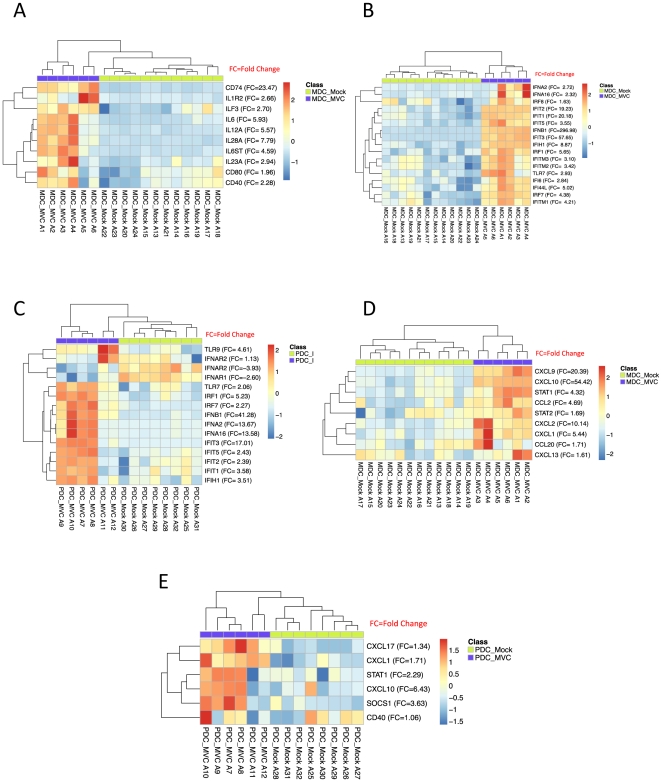
Significant genes associated with specific immune function in MVA-C-infected mDCs and pDCs. A) Gene expression heatmap using selected set of immune related genes resulting from comparing myeloid DCs infected with MVA-C (MDC_MVC) and mock-infected (MDC_Mock) groups. B) Gene expression heatmap using selected set of IFN-induced genes resulting from comparing myeloid DCs infected with MVA-C (MDC_MVC) and mock-infected (MDC_Mock) groups. C) Gene expression heatmap using selected set of IFN signaling genes resulting from comparing plasmacytoid DCs infected with MVA-C (PDC_MVC) and mock-infected (PDC_Mock) groups. D) Gene expression heatmap using selected set of inflammation associated genes resulting from comparing myeloid DCs infected with MVA-C (MDC_MVC) and mock-infected (MDC_Mock) groups. E) Gene expression heatmap using selected set of inflammation associated genes resulting from comparing plasmacytoid DCs infected with MVA-C (PDC_MVC) and mock-infected (PDC_Mock) groups. The genes selected are differentially expressed (FC>1.3 and adjusted p-value<0.05). Red and Blue correspond to up- and down-regulation respectively.

In order to characterize the global response elicited by MVA-C in *ex vivo* purified human myeloid (mDCs) and plasmacytoid (pDCs) dendritic cells, we determined the transcriptional profiles using gene array analysis. We compared the transcriptional profiles of MVA-C infected mDCs and pDCs (n = 6) to mock infected cells (n = 14). Unsupervised clustering using multidimensional scaling (MDS) revealed, as expected, distinct transcriptional profiles between mDCs and pDCs either in the mock- or MVA-C-infected subsets (Fig. S1). This confirms our previous reports showing differences in the transcriptional signatures of mDCs and pDCs in response to viral infection [7]. We then examined separately the gene expression of mDC and pDC in response to MVA-C infection. We show that infection of MVA-C significantly induces the expression of more than 5500 genes in mDCs and 4500 genes in pDCs when compared to mock infection (adjusted p-value of <0.05 false discover rate FDR). Complete lists of all significant genes that are induced following MVA-C infection of mDCs and pDCs are presented in tables S1 and S2, respectively. Further analysis of the top 30 genes induced by MVA-C in mDCs revealed the increased expression of interferon (IFNB1) and IFN-induced genes (IFIT2, IFIT3, OASL, GBP4), inflammatory molecules (CXCL9, CXCL10, and TNF-α) and antiviral genes such as ZC3HAV1 that has been shown to have a potent anti-retroviral activity [17] (Fig. 2A). Similarly, in pDCs the top 30 genes showed the increased expression of IFN genes (IFNB1, IFNA2, IFNA16), IFN-induced genes (IFIT3) and anti-retroviral genes such as TRIM5 (Fig. 2B). We then analysed significant genes that are associated with specific immune function in mDCs and pDCs infected with MVA-C. Fig. 3A shows that MVA-C induced the expression of genes that are associated with DC maturation and activation including CD40, CD80 and cytokines that are critical for T cell and B cell responses including IL-6, IL12, and IL-28. Furthermore, we examined genes that are critical for antiviral activity. Fig. 3B shows significant induction of more than 15 genes that are associated with IFN response in mDCs. Similar induction was also observed in pDCs (Fig. 3C). In addition, infection with MVA-C induced the expression of genes associated with inflammatory response such as CXCL9 and CXCL10 in mDC and pDC (Fig. 3D and Fig. 3E, respectively).

Overall, these results demonstrate that MVA-C induces the expression of large number of biologically significant genes in mDC and pDC subsets. These induced genes play an important role in regulating adaptive immune response as well as in exerting antiviral effect.

**Figure 4:**
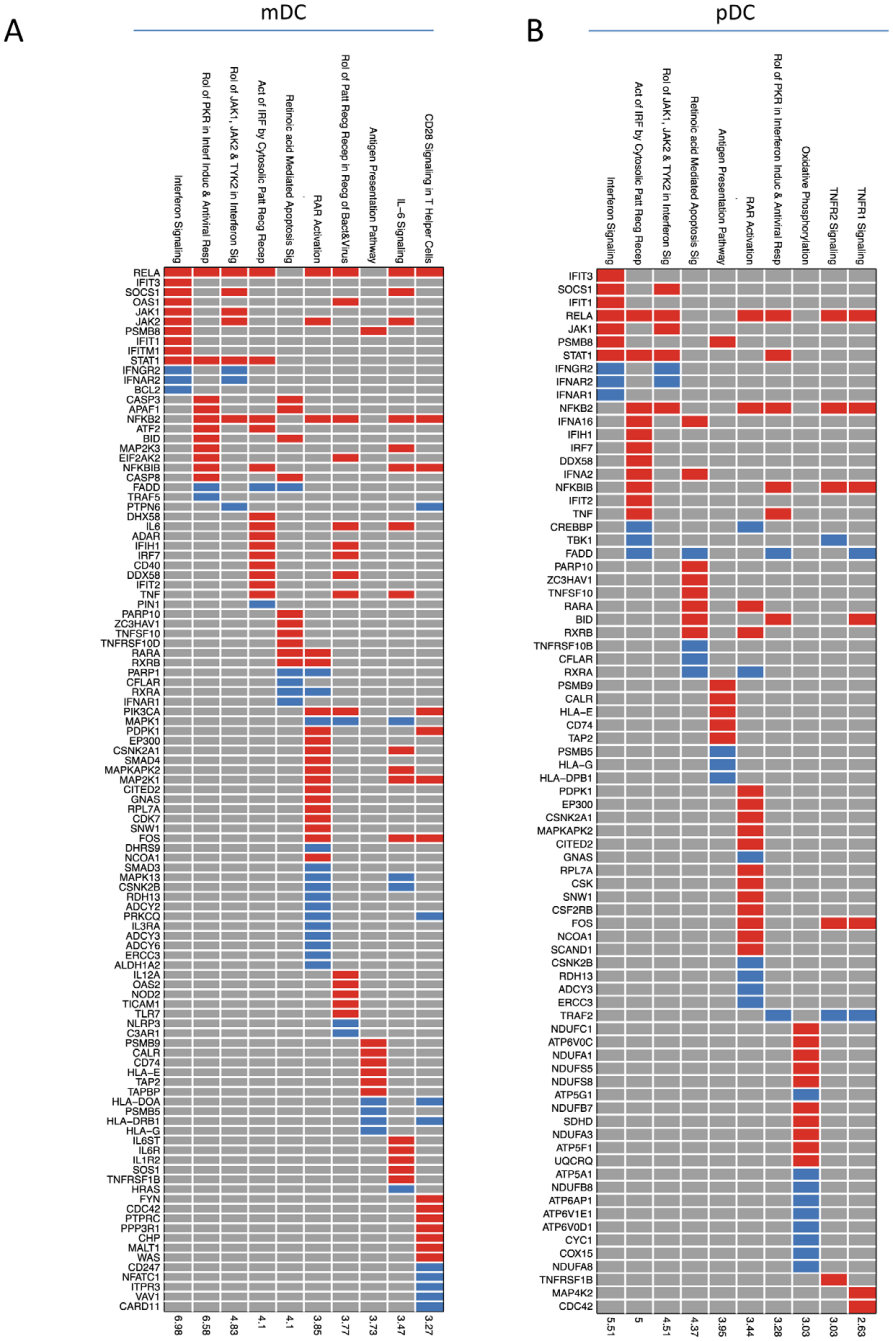
Signaling pathways induced upon MVA-C infection of mDCs and pDCs. Selected significant top 10 pathways and their genes members resulting from comparing myeloid DCs infected with MVA-C (MDC_MVC) and mock-infected (MDC_Mock) groups (A) or plasmacytoid DCs infected with MVA-C (PDC_MVC) and mock-infected (PDC_Mock) groups (B). Each row is an upregulated canonical pathway for innate immunity (Ingenuity software); each column represents an up- (red) or down- (blue) regulated gene (p<0.05 and |FC|>2) included in 1 or more regulated pathway(s). The over-representation test was performed using Fisher Exact Test and the significance, displayed on the right side, is achieved for p<0.05 (-log(p)>1.3).

While gene transcription does not provide functional information, however it gives an indication of how genes can be placed into specific cellular pathways. Thus, we investigated signaling pathways induced upon infection of mDC and pDC subsets with MVA-C. We observed the activation of multiple innate and adaptive immunoregulatory pathways in mDC and pDC compartments. Fig. 4A depicts a list of top signaling pathways that are elicited in mDC following infection with MVA-C. For example, MVA-C infection induces IFN signaling, antiviral and IRF pathways. Selective genes were depicted in each of these pathways including IFIT3, IFIT1, IFITM for IFN signaling; TRAF5, APAF1, IRF7 for antiviral, and ADAR, IRF7, IFIH1 and DDX58 for IRF pathways (Fig. 3). We also observed the induction of pathways associated with the activation of the adaptive immune response including CD28 signaling, IL-6 signaling, and antigen presentation. Similar innate and adaptive pathways were also induced in pDC infected with MVA-C (Fig. 4B).

### Maturation of DCs after infection with MVA-C

**Figure 5:**
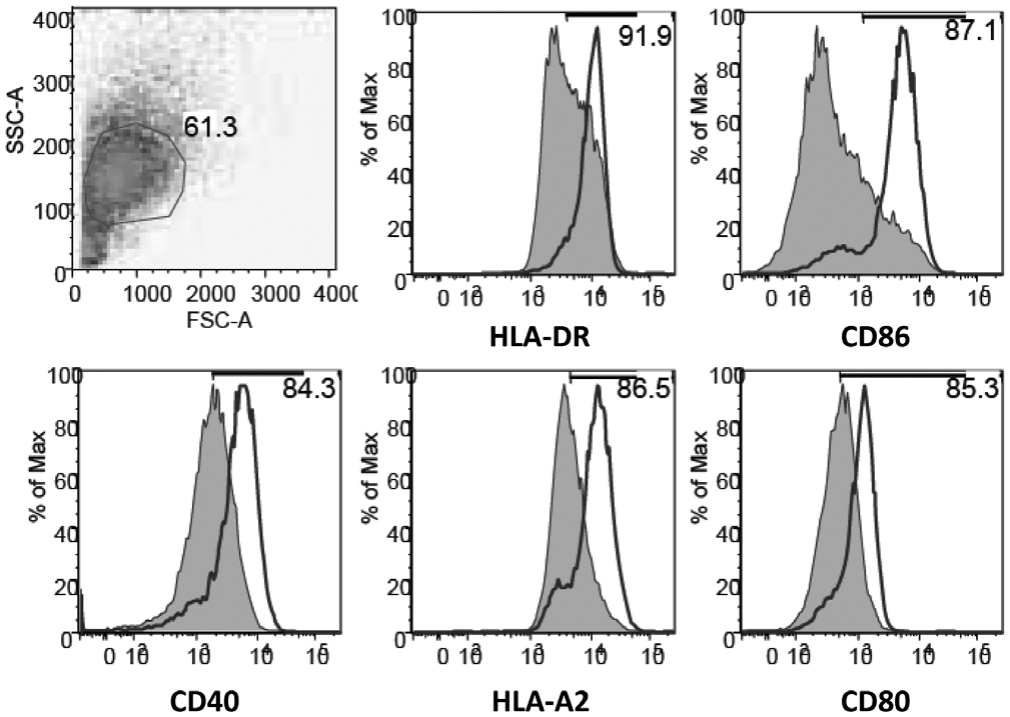
MVA-C induces maturation of human moDCs. Cells were infected with 5 pfu/cell and expression of different membrane surface markers was determined by flow cytometry at 24 h.p.i. The shaded graphs represent cells infected with a parental WT strain. Mean fluorescence intensity is indicated in the plots. Data are representative of at least five independent experiments.

Since previous studies have shown that infection of human DCs with the non-recombinant MVA leads to an increase in upregulation of CD86 and HLA-DR molecules [18], [19] and in order to confirm our gene expression data, we next determined the effects of MVA-C on moDC maturation through the analyses of several cellular surface markers. As shown in Fig. 5, expression levels of HLA-DR, CD86, CD40, HLA-A2 and CD80 were all increased in MVA-C-infected cells. These results demonstrate that MVA-C is able to induce enhanced phenotypic DC maturation in the absence of other stimuli.

### Enhanced cytokine and chemokine production after infection with MVA-C

**Figure 6:**
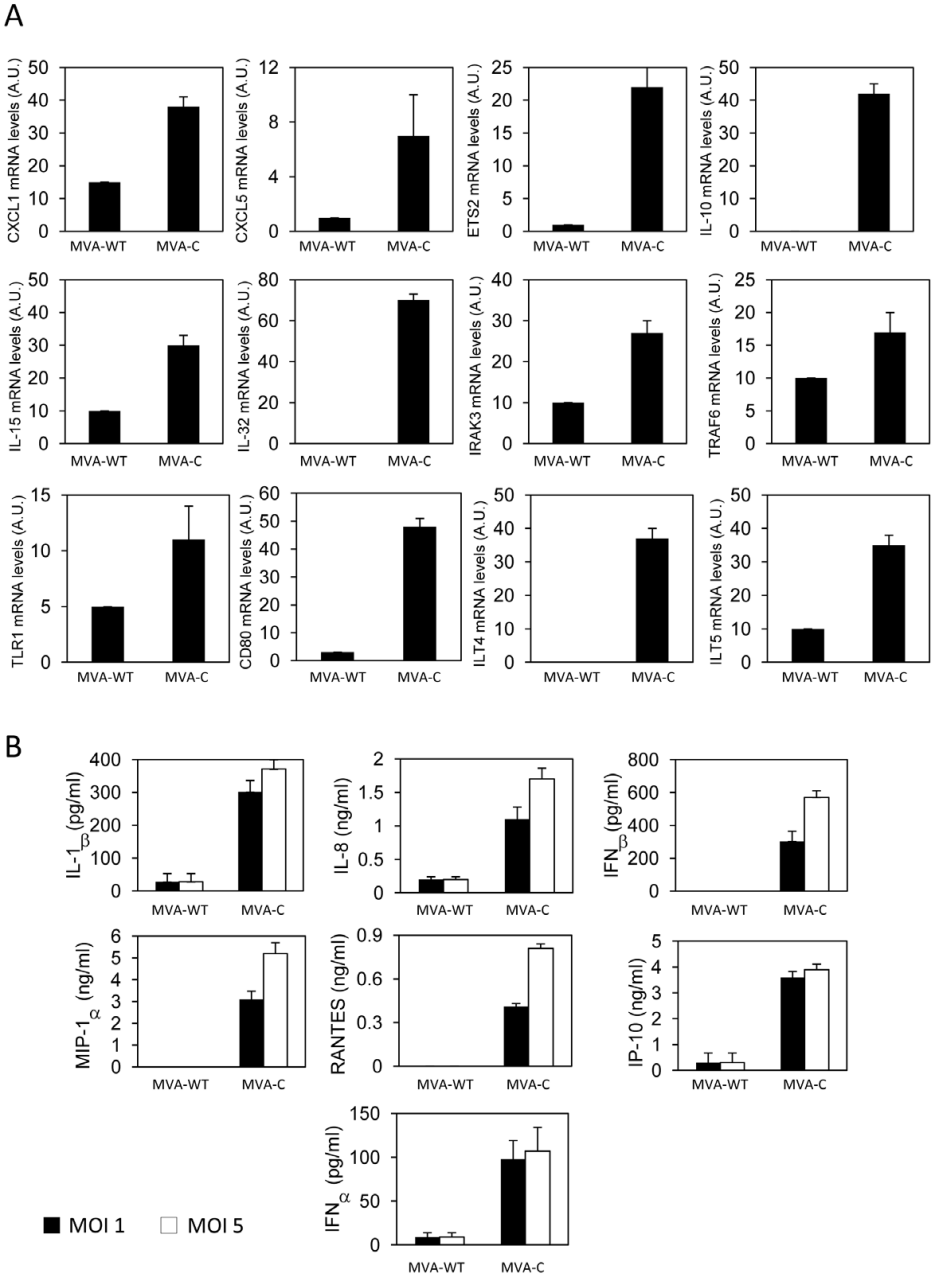
MVA-C induces cytokine and chemokine production in human primary macrophages. A) Human primary macrophages were infected at 5 pfu/cell with MVA-WT or MVA-C. At 3 h.p.i., cells were collected, RNA extracted and levels of different mRNAs were defined by RT-PCR. B) Human primary macrophages were infected (1 or 5 pfu/cell) with MVA-WT or MVA-C. Supernatants were collected at 24 h and used to quantify IL-1β, IL-8, IFNβ, MIP-1α, RANTES, IP-10 and IFNα by ELISA. Experiments were performed in triplicate samples from two human donors. Data are means and standard deviation from one experiment representative of at least two experiments.

To further characterize the effects of MVA-C infection on human cells, we next defined the expression levels of some selected cytokines, chemokines, signaling and accessory molecules in primary human macrophages. We first examined by RT-PCR the mRNA levels of these molecules in cells infected with MVA-C (5 pfu/cell) for 3 h. As shown in Fig. 6A, mRNA levels of genes encoding for chemokines (CXCL1 and CXCL5), cytokines (IL-10, IL-15 and IL-32), pattern recognition receptors (TLR1) and innate signaling molecules like IRAK3 and TRAF6, were all increased after MVA-C infection in comparison with MVA-WT-infected cells. Moreover, MVA-C induced the expression of CD80, ILT4, ILT5 and ETS2 mRNAs, suggesting an increased capacity to induce T cell signaling. Then, cell-free supernatants from primary human macrophages infected for 24 h with parental or recombinant MVA-C viruses (1 and 5 pfu/cell) were used to quantify by ELISA cytokine and chemokine production. As shown in Fig. 6B, there was an increase in the levels of type I IFNs (IFNα and IFNβ), chemokines (IP-10, MIP-1α and RANTES) and cytokines (IL-8 and IL-1β) secreted in the MVA-C-infected cells at the two different multiplicities compared with the control. Similar increases of chemokines and cytokines were obtained after MVA-C infection (24 h with 1 and 5 pfu/cell) of whole human blood (not shown).

### MVA-C infection induces cross-presentation to CD8 T cells and T cell proliferation

**Figure 7:**
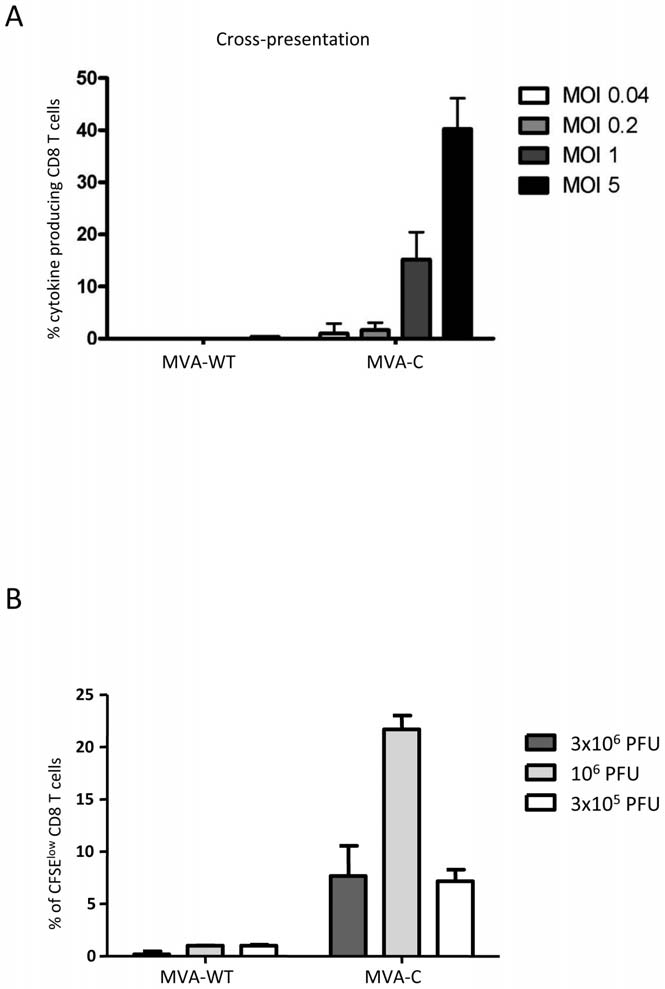
MVA-C infection induces antigen cross-presentation to CD8 T cells and T cell proliferation. A) Human moDCs were incubated with apoptotic infected-HeLa cells before a CD8 T cell clone was added. After overnight incubation, cells were harvested and among the lymphocyte population, CD8 cells were gated and analyzed for IFN-γ, TNF-α, IL-2 and MIP-1β production. Cytokine production by HIV-specific CD8 T cells was determined. Percentages of CD8 T cells producing any cytokine are indicated at the various virus multiplicities. B) MVA-C and parental MVA-WT were evaluated *in vitro* using cryopreserved PBMCs from an HIV-1-infected subject. Cell proliferation using the CFSE dilution assay was measured 6 days after stimulation. At the end of the stimulation period, cells were stained for CD3, CD4, CD8 and a viability marker and analyzed by flow cytometry. Mean values and standard deviation of at least three experiments are shown in panels A and B.

We have previously described an assay to determine the ability of moDCs to cross-present antigens from apoptotic infected HeLa cells [7]. Human moDCs were incubated with apoptotic infected HeLa cells before an HIV-specifc CD8 T cell clone was added and, after overnight incubation, cells were harvested and among the lymphocyte population, CD8 T cells were gated and analyzed for IFN-γ, TNF-α, IL-2 and MIP-1β production. The results shown in Fig. 7A with percentages of CD8 T cells producing any cytokine revealed cross-presentation to HIV-specific CD8 T cell clone triggered by MVA-C infection, in contrast with the control. This cross-presentation was virus multiplicity-dependent.

Furthermore, we also measured the HIV-specific proliferative capacity of CFSE-labeled human PBMCs from an HIV-infected long-term non-progressor upon infection with MVA-C or MVA-WT, as described under Materials and Methods. Fig. 7B represents CD8 T cell proliferation as determined by CFSE dilution measured at day 6 after stimulation with MVA-C at different virus multiplicities. High proliferation up to 20% was observed after infection with 10^6^ pfu of MVA-C. Together, the data of Fig. 7 revealed that both cross-presentation and proliferation of CD8 T cells are induced by MVA-C infection of human immune cells.

### MVA-C infection activates memory HIV-1-specific T cell immune responses

**Figure 8:**
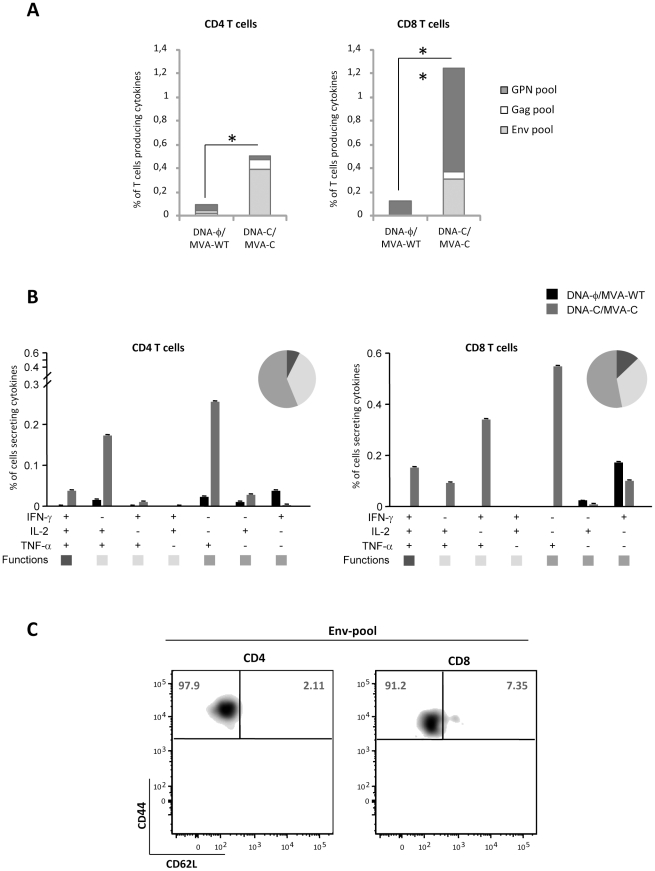
Long-lived memory immune response to HIV antigens elicited by MVA-C. A) Magnitude of vaccine-specific CD4^+^ and CD8^+^ T cells. The HIV-1-specific CD4 and CD8 T cells were measured 53 days after the last immunization by ICS assay following stimulation with the different HIV-1 peptide pools. The values represent the sum of the percentages of T cells secreting IFN-γ and/or TNF-α and/or IL-2 against Env+Gag+GPN peptide pools. All data are background subtracted. B) Functional profile of memory HIV-1-specific CD4 and CD8 T cell responses. All the possible combinations of the responses are shown on the *x* axis, whereas the percentage of the functionally distinct cell populations within the total CD4 or CD8 T cell populations are shown on the *y* axis. Responses are grouped and colour-coded on the basis of the number of functions. The non-specific responses obtained in the control group DNA-Φ/MVA-WT were subtracted in all populations. C) Phenotypic profile of memory HIV-1-specific CD4 or CD8 T cells. Representative FACS plots showing the percentage of Env-specific CD4 or CD8 T cells with central memory (TCM, CD44^high^CD62L^+^) or effector memory (TEM, CD44^high^CD62L^−^) phenotype. * p<0.05; ** p<0.005. p-values indicate significantly higher responses compared to DNA-Φ/MVA-WT immunization group.

Since triggering of both humoral and cellular arms of the immune system might be needed for HIV prevention in humans [20] and activation of T effector memory cell (TEM) response has been associated with protection in NHP model [21], [22], we next characterized the ability of MVA-C to activate HIV-1-specific immune responses in mice using a DNA prime/Pox boost approach. Thus, Balb/c mice were inoculated by intramuscular route (i.m.) with two plasmid DNA vectors expressing gp120 and GPN of clade C or with sham DNA and boosted two weeks later by intraperioneal route (i.p.) with MVA-C or parental MVA-WT. Vaccine-induced T cell immune responses were evaluated at 53 days after the booster by polychromatic ICS assay after the stimulation of splenocytes with a panel of 464 peptides (15 mers overlapping by 11 amino acids) grouped in three pools: Env (112 peptides), Gag (121 peptides) and GPN (231 peptides). The peptides encompassed the Env, Gag, Pol, and Nef proteins of HIV-1 and were designed based on the sequence of the immunogens expressed by MVA-C. As shown in Fig. 8A, the magnitude of the HIV-1-specific CD4^+^ and CD8^+^ T cell responses, determined as the sum of the individual responses obtained for Env, Gag and GPN peptide pools, was significantly higher in mice that received DNA-C prime/MVA-C boost than that obtained in the control group DNA-φ/MVA-WT (p<0.05). The CD4^+^ T cell responses were mainly directed against the Env pool whereas the CD8^+^ T cell responses were higher in magnitude, and broader, triggered by both Env and GPN pools. On the basis of the analysis of IL-2, TNF-α and IFN-γ secretion, seven distinct HIV-1-specific CD4^+^ and CD8^+^ T cell populations were identified (Fig. 8B). Vaccine-induced CD4^+^ and CD8^+^ T cell responses were polyfunctional, with more than 40% of antigen-specific T cells exhibiting two or three functions. We also determined the phenotype of the memory responses by measuring the expression of CD62L and CD44 in the HIV-1-specific T cells. The effector memory T cells (TEM) have a CD44^high^CD62L^−^ phenotype whereas the central memory T cells (TCM) are CD44^high^CD62L^+^. As shown in Fig. 8C, the HIV-1-specific CD4 and CD8 T cells have a TEM phenotype, since more than 90% of the responding cells were CD44^high^CD62L^−^. Similar findings were observed in two independent experiments. Overall, the results revealed that MVA-C positively impacts on the CD4^+^ and CD8^+^ T cell memory phase of the immune response.

### MVA-C induces humoral response to Env

**Figure 9:**
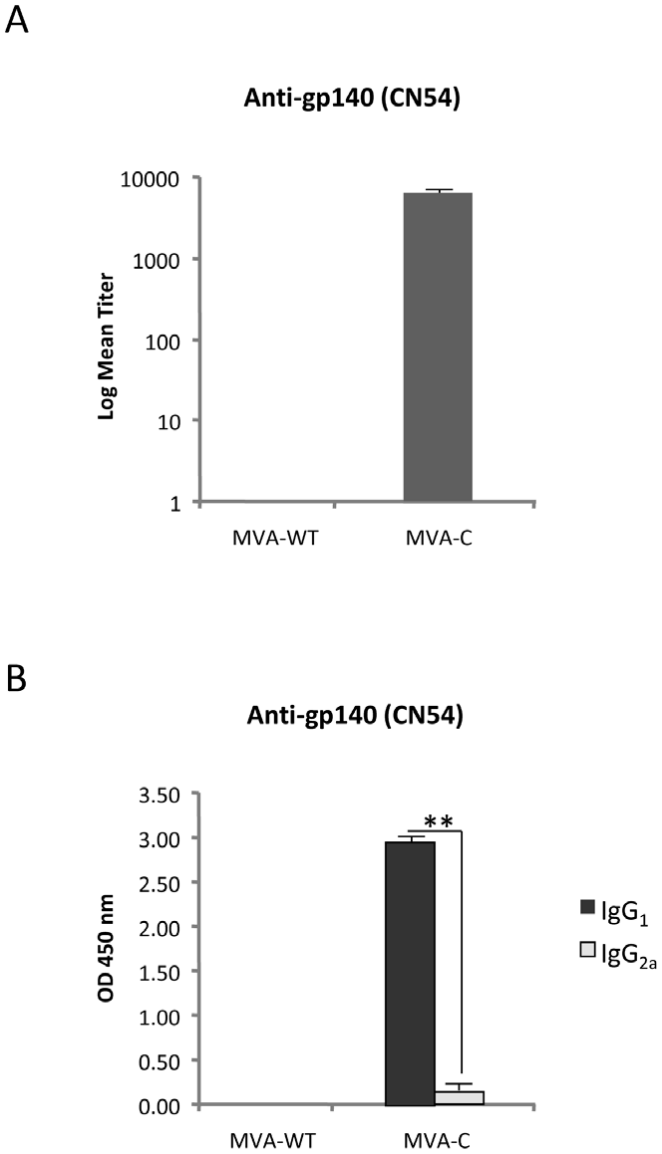
Humoral immune responses elicited by MVA-C against HIV-1 gp140 protein. Levels of Env-specific IgG binding antibodies (A) or IgG subtypes (B) were measured in serum from naïve and immunized mice at 53 days after the boost. The values represent the mean antibodies titer for each group. ** p<0.005.

Since induction of antibodies to gp120 has been associated with a reduced risk of HIV infection in humans [23], we next evaluated the antibody levels in serum from mice vaccinated in a DNA prime/MVA boost protocol. An ELISA test with plates coated with purified gp140 CN54 protein was used for antibody specificity. As shown in Fig. 9A, anti-gp140 IgG binding antibodies were detected only in animals that received DNA-C prime/MVA-C boost, indicating that the released gp120 expressed during infection with MVA-C triggered specific humoral responses. Almost all the anti-Env IgG binding antibodies detected in MVA-C immunized animals were of IgG_1_ subtype (Fig. 9B), indicating a Th2 response.

## Discussion

At present, there is limited number of attenuated poxvirus vectors expressing several HIV antigens considered as candidate vaccines against HIV/AIDS that have entered phase I/II/III clinical trials. The most advanced poxvirus vector with an attenuated phenotype is the canarypox ALVAC expressing gp120/Gag-Pro that has shown partial efficacy when combined with the protein gp120 in the phase III clinical trial in Thailand [1]. Other attenuated poxvirus vectors like NYVAC [9], [24], MVA [12], [25], [26], [27], [28], [29], [30] or Fowlpox [31] have shown different immunogenic profiles when used alone or in combination with other heterologous vectors in clinical trials, probably due the nature of the vector, virus dose and HIV antigens being expressed. We have previously shown that when poxvirus vectors MVA and NYVAC expressing gp120/Gag-Pol-Nef are compared head-to-head, there are clear differences in the induction of cellular genes in response to virus infection that might lead to differences in the activation of host cell immune responses [14], [32]. Hence, the immune response induced by each single poxvirus vector strain must take into account those observations, as the immunological properties might differ. In addition, replication competent poxvirus vectors are also sought as vaccine candidates that might be able to enhance the specific HIV immune responses, an aspect that will be known in future clinical trials. From the point of view of attenuated MVA vectors, many different vaccine candidates expressing diverse HIV antigens have moved into clinical trials with encouraging results [12], [25], [26], [27], [28], [29], [30]. We have recently described the results of a phase I clinical trial with an MVA vector expressing gp120 and Gag-Pol-Nef of HIV-1 from clade B (MVA-B). We showed that MVA-B was safe, well tolerated and highly immunogenic, inducing broad, polyfunctional, and long-lasting CD4^+^ and CD8^+^ T cell responses to HIV-1 antigens, with preference for effector memory T cells. Moreover, the vaccination regimen also induced antibody responses to Env in the 95% of volunteers [26], [27].

As a new step to move forward with an MVA candidate for clade C, the most prevalent in parts of Africa and Asia, here we have performed an in depth characterization of the biological and immune properties of the MVA-C vaccine vector that will enter soon in phase I clinical trials. We showed that MVA-C is an excellent immunogen, since it expresses at high levels the HIV antigens in human moDCs, triggers DC maturation, activates the expression of immunostimulatory molecules, induces cross-priming and CD8^+^ T cell proliferation and, in mice vaccinated by prime/boost protocol, induces antibodies and activates HIV-1-specific CD4 and CD8 T cell memory responses that are polyfunctional and of effector memory phenotype. In the context of HIV vaccines, these biological and immune characteristics might be relevant in the control of an HIV infection. In fact, it is generally assumed that both humoral and cellular arms of the immune system need to be activated in order to control HIV infection, either through neutralizing antibodies, ADCC, or cytotoxic T cells [20]. From the Thai trial and after examining a large population of vaccinated individuals, it has been suggested that production of antibodies, specifically directed to the V2 loop, might be needed to control HIV infection, while high levels of IgA are detrimental [23]. In the Thai trial the T cell responses were low and, hence, it has not been as yet possible to conclude that this arm of the immune system is dispensable or required. In fact, from the NHP studies with a cytomegalovirus vector expressing SIV antigens, it has been proposed that immune protection against a virulent SIV challenge required activation of T cells of an effector memory phenotype (TEM) [21], [22]. In view of the fact that, as shown here, MVA-C activates both *in vitr*o and *in vivo* different components of the immune system that might be needed for protection against a pathogen, our immunogenicity study in mice establish that MVA-C is an excellent HIV vaccine candidate with production of both antibodies and T cell responses to HIV antigens. It is significant the polyfunctional and broad nature of the immune response triggered in mice, with preferential activation in a DNA-C prime/MVA-C boost of memory T cell responses of TEM phenotype. The vaccine-induced CD4^+^ T cell response was preferentially against Env whereas the CD8^+^ T cell response was directed against GPN and Env antigens. This is particularly important when considering vaccination protocols, as multiple challenges with the same immunogen might not be required. Future animal studies should elucidate the optimal combination of MVA-C with other vectors (i.e., Env protein, heterologous viral vectors, adjuvants) that trigger enhanced B and T cell responses. Overall, the results reported here points MVA-C vector as promising HIV/AIDS vaccine candidate and support the future phase I clinical trials with MVA-C.

## Materials and Methods

### Ethics statement

The animal studies were approved by the Ethical Committee of Animal Experimentation (CEEA-CNB) of Centro Nacional de Biotecnologia (CNB-CSIC) in accordance with national and international guidelines and with the Royal Decree (RD 1201/2005). Permit numbers: 152/07 and 080030.

### Cells and viruses

Monocyte derived dendritic cells (moDCs) were obtained from cryopreserved or freshly isolated peripheral blood mononuclear cells (PBMCs) from buffy coats of healthy blood donors. CD14^+^ monocytes were isolated from PBMCs by positive selection with CD14 microbeads (Miltenyi Biotech, Bergisch Gladbach, Germany). The obtained monocytes were plated at 1×10^6^ cells/ml and subsequently cultured with GM-CSF (800 U/ml) and IL-4 (500 U/ml) for 5 days to differentiate into moDCs as described previously [7]. Fresh medium containing GM-CSF and IL-4 was added at day 2. Human myeloid DCs (mDCs) and plasmacytoid DCs (pDCs) were obtained from freshly isolated PBMCs by positive selection as previously described [7]. Purity of sorted DC populations was over 99%.

Macrophages were obtained by culturing adherent PBMCs cells for 6 days in RPMI 1640 with Glutamax (Invitrogen) and 10% heat-inactivated FCS (Sigma-Aldrich). Human whole blood assay was performed as described [33].

HeLa cells (ATCC, Manassas, VA) were cultured in IMDM containing 8% fetal bovine serum (PAA) and 80 IU/ml Natrium-penicillin (Astellas Pharma).

HIV-specific CD8 T cells were obtained from an HIV-1 seropositive long-term non-progressor [7]. First, total PBMCs were depleted for CD4 T cells using CD4 dynabeads (Dynal) according to the manufacturer's protocol. The enriched CD8 T cell population was subsequently treated as previously described [7]. Specificity was confirmed after 4 weeks of culture. Although these CD8 T cells are not cloned from a limiting dilution, 99.8% of the T cells express the Vβ22 TCR, suggesting that these cells are obtained from a single precursor and can be considered clonal. Cells were restimulated every two weeks. Cells were left untreated for at least two weeks before use in antigen presentation assay.

Primary chicken embryo fibroblast cells (CEF) and DF-1 cells (a spontaneously immortalized chicken embryo fibroblast cell line; ATCC, Manassas, VA) were grown in Dulbecco's modified Eagle's medium (DMEM) supplemented with 10% fetal calf serum (FCS).

All cells were maintained in a humidified air 5% CO_2_ atmosphere at 37°C except DF-1, that were maintained at 39°C.

MVA-C, a recombinant vaccinia virus Ankara expressing in the TK locus the HIV-1 clade C Gag, Pol, Nef and Env antigens (Fig. 1A), was constructed by homologous recombination in CEF cells. Gag-Pol-Nef is a fusion protein composed of *gag, pol* and *nef* ORFs from HIV-1 clone CN54, which has been modified to enhance its immunogenicity and removed, for safety considerations, undesirable domains of the HIV antigens. Gp120 Env protein belongs to the same HIV-1 isolate (CN54). In both cases, the codon usage was adapted to highly express human genes. The coding sequence, gene organization, generation and some of the immunological properties of MVA-C have been described previously [15]. The parental and recombinant MVA-C viruses were grown in CEF cells, purified through two 36% (w/v) sucrose cushions, and titrated by plaque immunostaining assay in DF-1 cells as previously described [34].

### HIV-1 *gag* expression

The expression of *gag* protein was measured in moDCs at different times post-infection as previously described [7]. Cells were infected for 1 h at MOI 1 and subsequently washed thoroughly. From 2 to 24 h of incubation, cells were harvested and *gag* expression was determined by intracellular staining with an anti-*gag* specific antibody (KC57, Beckman Coulter). Cells were analyzed on a FACSCalibur using CellQuest (BD). FACS data were analyzed with FlowJo (Tree Star, Inc.).

### Gene array analysis

Isolated mDCs and pDCs subsets were infected with MVA-C or mock-infected for 6 h before cells were harvested for gene expression profiling as previously described [7]. Total RNA was purified from DC subsets using DCsRNA extraction kits (Qiagen,USA). Quantification was performed using a spectrophotometer (NanoDrop Technologies, Willington, DE) and RNA quality was assessed using the Experion™ Automated Electrophoresis System (Bio-Rad, Hercules, CA). Total RNA was then amplified and labeled using the Illumina® TotalPrep™ RNA Amplification kit (Illumina, Inc., San Diego, CA) as previously described [7]. The biotinylated cRNA was then hybridized onto Illumina Human RefSeq-8 V2 and V3 BeadChips expressing more than 24000 probe sets spanning the whole human genome (PBMC samples) and quantified using Illumina BeadStation 500GX scanner and Illumina BeadScan software.

Illumina probe data were exported from BeadStudio as raw data and were screened for quality; samples failing chip visual inspection and control examination were removed. Probeset from the two Illumina platforms were mapped to a common probeset Id using a mapping file provided by Illumina. A dataset containing probeset common to both platforms was then used for subsequent steps. Gene expression data was preprocessed.

Analysis was conducted using the R statistical language (R Development Core Team) and various software packages from Bioconductor, an open source project for the analysis and comprehension of high-throughput genomic data [35]. First, arrays displaying unusually low median intensity, low variability or low correlation relative to the bulk of the arrays were discarded from the rest of the analysis. Quantile normalization was applied, followed by a log_2_ transformation. Bioconductor's gene filter package was then used to filter out genes with low expression and insufficient variation in expression across all samples tested. Expression values retained after this filtering process presented intensities greater than 80 units in at least 2 samples and a log base 2 scale of at least 0.3 for the interquartile range (IQR) across all samples tested. The LIMMA package from Bioconductor [36] was used to fit a linear model to each probe and to perform a (moderated) t-test on various differences of interest. The expected proportions of false positives (FDR) were estimated from the unadjusted p value using the Benjamini and Hochberg method [37].

### Pathway analysis

Ingenuity Pathway Analysis software (Ingenuity® Systems, www.ingenuity.com) was used to identify canonical signaling pathways regulated by mDCs infected with MVA-C (MDC_MVC) or pDCs infected with MVA-C (PDC_MVC). Canonical pathway analysis identified the pathways from the Ingenuity Pathway Analysis library of canonical pathways that were most significant to the dataset. Illumina Probe IDs were imported into the Ingenuity software and mapped to the Gene Symbol from Ingenuity database. Genes that had adjusted p-value<0.05 and |FC|>2 and associated with a canonical pathway in Ingenuity's Knowledge Base were used for pathway analysis. Over-representation Fisher's exact test was used to calculate a p-value determining the probability that the association between the genes in the dataset and the canonical pathway is explained by chance alone. The pathways were ranked by -log p-value. This score was used as the cut-off for identifying canonical pathways significantly (p value<0.05).

### Infection of DCs and flow cytometry

Freshly isolated moDCs were infected with the different viruses at MOI 5 [7]. After 1 h of incubation, the cells were washed extensively and plated into 24-well plates. Cells were harvested at 24 h.p.i. and fixed in 4% paraformaldehyde and subsequently incubated with the following antibodies: α-CD86 PE-Cy5 (clone IT2.2), α-CD80 PE-Cy5 (clone 2D10.4), α-CD11c Alexa Fluor 700 (clone 3.9) (all from eBiosciences), α-CD40 APC, α-HLA-A2 FITC and α-HLA-DR PE (all from Becton Dickinson). Cells were analyzed on a LSRII flow cytometer using DIVA (BD). FACS data were analyzed with FlowJo.

### RNA analysis by quantitative real-time polymerase chain reaction

Total RNA was isolated from primary human macrophages using the RNeasy kit (Qiagen). Reverse transcription and real-time PCR (RT-PCR) was performed with a 7500 Fast Real-Time PCR System (Applied Biosystems, Rotkreuz, Switzerland) using the Power SYBR Green PCR Master Mix (Applied Biosystems) and primer pairs previously described [33]. All samples were tested in triplicates. Gene specific expression was expressed relative to the expression of *HPRT* in arbitrary units (AU). Gene specific over *HPRT* ratios were validated using the house-keeping gene *ACTB*.

### Measurement of cytokine and chemokine production

The concentrations of human IL-1β (Bender MedSystems, Vienna, Austria), IL-8 (BD Biosciences), RANTES, IP-10, MIP-1α (R&D), IFNα and IFNβ (PBL Biomedical Laboratories, Piscataway, NJ) in whole blood assay and in cell culture supernatants were measured by ELISA as previously described [33].

### Antigen presentation assays

Antigen presentation to HIV-specific CD8 T cells was studied using moDCs cross-presenting antigens from HeLa cells that were infected at different MOIs. In addition, the cytokine production of HIV-specific CD8 T cells was assessed as previously described [7]. For that, HeLa cells were harvested by EDTA and infected with MVA-C or MVA-WT at different MOIs for 1 h. Cells were extensively washed to remove residual virus. After overnight incubation, cells were irradiated with UV-C (200 µW/cm^2^) to ensure that no residual virus and no viable cells were present and thus exclude direct presentation. Apoptotic virus-infected HeLa cells were harvested and added to moDCs at a 2∶1 ratio. After 6 h incubation, HIV- or VACV-specific CD8 T cells were added (at approximately 5 T cells : 1 DC ratio) followed by overnight culture at 37°C/5%CO_2_. Brefeldin A (10 µg/ml, Sigma-Aldrich) was added to retain cytokines within the T cells allowing the detection of multiple cytokines. After 18 hours, intracellular cytokine staining (ICS) was performed as described. Cells were fixed and permeabilized using Cytofix/Cytoperm™ Fixation/Permeabilization Solution Kit (BD). Cells were then incubated with α-TNF PE-Cy7 (clone MAb11, eBiosciences), α-IFN-γ FITC, α-IL-2 APC, α-MIP-1β PE (all three from BD) and α-CD8 PerCP (Dako) antibodies. After washing, cells were analyzed on a LSRII flow cytometer using DIVA (BD). FACS data were analyzed with FlowJo. Net accumulation is the percentage of live CD8^+^ cells expressing a specific cytokine upon stimulation with moDCs loaded with apoptotic virus-infected HeLa cells.

### *Ex vivo* proliferation assay

Overnight-rested cryopreserved PBMCs from an HIV-1 seropositive long-term non-progressor were washed twice, resuspended at 1×10^6^ cells/ml in PBS and incubated for 7′ at 37°C with 0.25 µM 5,6-carboxyfluorescein succinimidyl ester (CFSE, Molecular Probes, USA) as described [38]. Then, the reaction was quenched with one volume of FCS and cells were washed twice. Cells were then cultured (1×10^6^ cells in 1 ml of complete medium) in the presence of MVA-WT or MVA-C vectors at different MOIs, medium alone (negative control) or Staphylococcal enterotoxin serotype B (SEB, 40 ng/ml, positive control). At day 6, cells were harvested, stained for dead cells using the Aqua LIVE/DEAD stain kit (Invitrogen) and then with anti-CD3, -CD4 and -CD8 antibodies. After fixation, cells were acquired on an LSRII flow cytometer using DIVA (BD). FACS data were analyzed with FlowJo (8.8.2). The number of lymphocyte-gated events ranged between 1×10^5^ and 5×10^5^ in all experiments.

### Mice immunization schedule

Balb/c mice (6–8 weeks old) were purchased from Harlan. DNA prime/Poxvirus boost immunization protocol was performed as previously described [15]. Groups of animals (n = 4) received 100 µg of DNA-C (50 µg of pcDNA-_CN54_gp120 +50 µg of pcDNA-_CN54_GPN) by intramuscular route (i.m.) and two weeks later received an intraperitoneal (i.p.) inoculation of 1×10^7^ pfu of the corresponding virus. Animals primed with sham DNA (DNA-φ) and boosted with the non-recombinant MVA-WT were used as control group. At 53 days after the last immunization, animals were sacrificed and spleens processed for Intracellular Cytokine Staining (ICS) assays to measure the HIV-1-specific memory immune responses. Two independent experiments have been performed for the different groups.

### Intracellular cytokine staining assay (ICS)

The magnitude, polyfunctionality and phenotype of the HIV-1 specific T cell responses were analyzed by ICS. After an overnight rest, 5×10^6^ splenocytes (depleted of red blood cells) were stimulated during 6 h in complete RPMI 1640 media containing 1 µl/ml Golgiplug (BD Biosciences) and 5 µg/ml of different HIV-1 peptide pools that have being previously described [15]. At the end of the stimulation period, cells were washed, stained for the surface markers, permeabilized (Cytofix/Cytoperm kit; BD Biosciences) and stained intracellularly using the appropriate fluorochromes. For functional analyses the following fluorochromes-conjugated antibodies were used: CD3-FITC, CD4-Alexa 700, CD8-PerCP or −V500, IL-2-PE or -APC, IFN-γ-APC or -PECy7 and TNF-α-PECy7 or −PE. In addition, for phenotypic analyses the following antibodies were used: CD62L-FITC and CD44-SPRD. Dead cells were excluded using the violet LIVE/DEAD stain kit (Invitrogen). All antibodies were from BD Biosciences. Cells were acquired using an LSRII flow cytometer (BD Immunocytometry Systems). Analyses of the data were performed using the FlowJo software version 8.5.3 (Tree Star, Ashland, OR). The number of lymphocyte-gated events ranged between 1×10^5^ and 1×10^6^. After gating, Boolean combinations of single functional gates were then created using FlowJo software to determine the frequency of each response based on all possible combinations of cytokine expression or all possible combinations of differentiation marker expression. Background responses detected in negative control samples were subtracted from those detected in stimulated samples for every specific functional combination.

### Antibody measurements by ELISA

Binding antibodies to Env protein in serum were assessed by ELISA as previously described [15]. Serum from naïve and immunized mice were serially two-fold diluted in duplicate and reacted against 2 µg/ml of the recombinant 97CN54 gp140 purified protein (clade C) (kindly provided by Simon A. Jeffs, Imperial College London, UK). The antibody titer of Env-specific IgG was defined as the last dilution of serum that gives 3 times the mean OD450 value of the naïve control. For analysis of IgG subtypes, serum from naïve and immunized mice were reacted at 1∶100 dilution in triplicate against 2 µg/ml of the recombinant 97CN54 gp140.

### Statistical analyses

For antigen presentation and *ex-vivo* proliferation assays the p-values were calculated using Mann-Whitney U test using SPSS 16.0 (SPSS Inc). For RNA levels and cytokine production the comparisons among treatment groups were performed by two-tailed paired Student's *t*-test. Previously, a normality test was used to verify that samples assumed normal distribution. p-values less than 0.05 were considered to indicate statistical significance.

For the statistical analysis of ICS data we used a novel approach that corrects measurements for the medium response (RPMI) and at the same time allows the calculation of confidence intervals and p-values of hypothesis tests [39], [40]. The background for the different cytokines in the unstimulated controls never exceeded 0.05%. The data analysis program, Simplified Presentation of Incredibly Complex Evaluations (SPICE, version 4.1.5, Mario Roederer, Vaccine Research Center, NIAID, NIH), was used to analyze and generate graphical representations of T cell responses detected by polychromatic flow cytometry. All values used for analyzing proportionate representation of responses are background-subtracted.

## Supporting Information

Figure S1 **Multidimensional scaling plot illustrating distinct gene expression profile of the mDCs and pDCs infected with MVA-C (MDC_MVC and PDC_MVC) and mock-infected groups (MDC_Mock and PDC_Mock).** 8552 probes that pass the filtering step were used for this plot.
(TIF)

Table S1 List of differentially expressed (up- or down-regulated) genes between myeloid DCs infected with MVA-C (MDC_MVC) and mock-infected (MDC_Mock) groups. The threshold of differential expression is set at FDR 5%, with FC>1.3.
(XLS)

Table S2 List of differentially expressed (up- or down-regulated) genes between plasmacytoid DCs infected with MVA-C (PDC_MVC) and mock-infected (PDC_Mock) groups. The threshold of differential expression is set at FDR 5%, with FC>1.3.
(XLS)
