# Correction: Systems Analysis of MVA-C Induced Immune Response Reveals Its Significance as a Vaccine Candidate against HIV/AIDS of Clade C

**DOI:** 10.1371/annotation/09c18b10-8433-4d64-be1c-61e578cc088b

**Published:** 2012-05-17

**Authors:** Carmen Elena Gómez, Beatriz Perdiguero, Victoria Jiménez, Abdelali Filali-Mouhim, Khader Ghneim, Elias K. Haddad, Esther D. Quakkerlaar, Julie Delaloye, Alexandre Harari, Thierry Roger, Thomas Dunhen, Rafick P. Sékaly, Cornelis J. M. Melief, Thierry Calandra, Federica Sallusto, Antonio Lanzavecchia, Ralf Wagner, Giuseppe Pantaleo, Mariano Esteban

The seventh author's last name is misspelled. The author's correct full name is Esther D. Quakkelaar.

