# Correction: Systems Analysis of MVA-C Induced Immune Response Reveals Its Significance as a Vaccine Candidate against HIV/AIDS of Clade C

**DOI:** 10.1371/annotation/44b8f849-7885-4ad0-8fc9-b252c9ee1790

**Published:** 2012-09-17

**Authors:** Carmen Elena Gómez, Beatriz Perdiguero, Victoria Jiménez, Abdelali Filali-Mouhim, Khader Ghneim, Elias K. Haddad, Esther D. Quakkerlaar, Julie Delaloye, Alexandre Harari, Thierry Roger, Thomas Duhen, Rafick P. Sékaly, Cornelis J. M. Melief, Thierry Calandra, Federica Sallusto, Antonio Lanzavecchia, Ralf Wagner, Giuseppe Pantaleo, Mariano Esteban

The eleventh author's last name was spelled incorrectly. The correct name is: Thomas Duhen.

