# Supplementary figures and images for: Systems Analysis of MVA-C Induced Immune Response Reveals Its Significance as a Vaccine Candidate against HIV/AIDS of Clade C
Source: PLoS One. 2012 Apr 19;7(4):e35485. doi: 10.1371/journal.pone.0035485 (PMC3334902; doi:10.1371/journal.pone.0035485)

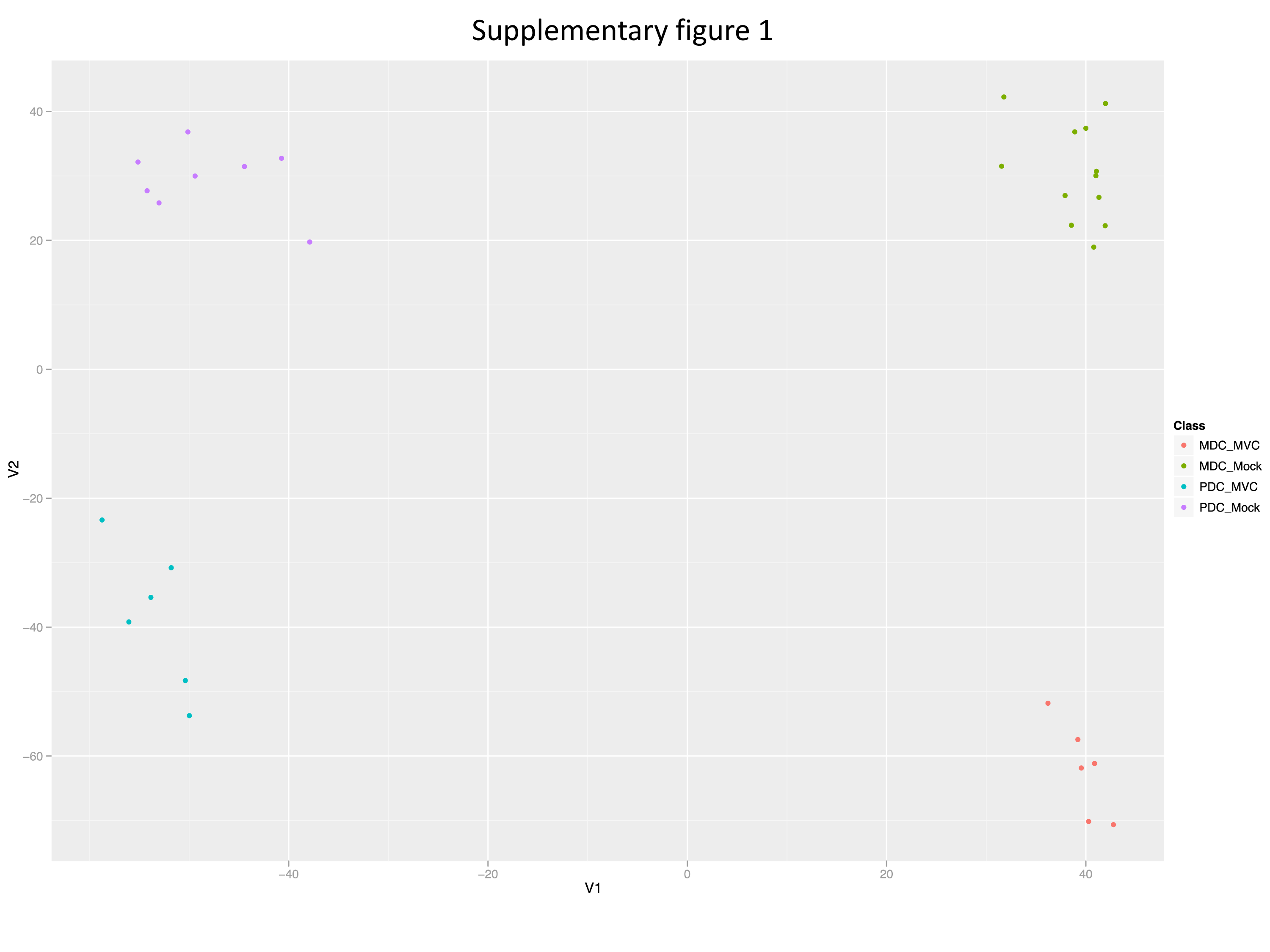

Supplement: Figure S1 — Multidimensional scaling plot illustrating distinct gene expression profile of the mDCs and pDCs infected with MVA-C (MDC_MVC and PDC_MVC) and mock-infected groups (MDC_Mock and PDC_Mock). 8552 probes that pass the filtering step were used for this plot. (TIF) [file pone.0035485.s001.tif]
